# Effects of the pandemic and economic crisis on public search trends related to oral and maxillofacial surgery in Türkiye: a Google Trends time-series analysis (2020–2025)

**DOI:** 10.1186/s12903-026-07878-7

**Published:** 2026-02-13

**Authors:** Berrin İyilikci

**Affiliations:** Department of Oral and Maxillofacial Surgery, Faculty of Dentistry, Pamukkale University, Denizli, Türkiye

**Keywords:** Google Trends, Oral and maxillofacial surgery, Pandemic, Economic crisis, Digital epidemiology

## Abstract

**Background:**

Large-scale societal disruptions, including pandemics and economic crises, profoundly influence healthcare-seeking behavior. Oral and maxillofacial surgery (OMS) comprises both essential and elective procedures and is therefore particularly sensitive to external pressures. This study investigated how the COVID-19 pandemic and subsequent economic downturn affected public interest in OMS-related procedures in Türkiye via Google Trends.

**Methods:**

This observational digital epidemiology time series study analyzed weekly relative search volume (RSV) data for four OMS-related keywords (“wisdom tooth extraction,” “oral surgery,” “implant,” and “jaw surgery”) retrieved from Google Trends between January 2020 and June 2025. Societal periods were categorized as pandemic restrictions/recovery, transition/normalization buffers, economic downturns, and extended follow-up periods. Data normality was assessed via the Shapiro–Wilk test. Between-period comparisons were conducted via the Kruskal–Wallis test with Dunn’s post hoc pairwise comparisons (Holm–Bonferroni adjustment). Temporal changes were evaluated using segmented regression (interrupted time series analysis), enabling the assessment of level and trend changes across predefined societal periods with robust Newey–West standard errors.

**Results:**

Interest in “wisdom tooth extraction” and “oral surgery” increased significantly during the postpandemic recovery period (*p* < 0.05), which is consistent with the rebound demand for deferred essential care. In contrast, interest in “implant” and “jaw surgery” declined significantly during the economic downturn (*p* < 0.01), indicating reduced public attention to high-cost elective procedures due to financial constraints.

**Conclusions:**

Public online interest in OMS procedures in Türkiye was shaped differently by pandemic-related service disruptions and subsequent economic pressures. Google Trends appears to be a valuable complementary tool for monitoring population-level shifts in healthcare interest and may support proactive clinical planning and health policy decision making during periods of societal instability.

**Supplementary Information:**

The online version contains supplementary material available at 10.1186/s12903-026-07878-7.

## Background

Oral and maxillofacial surgery (OMS) encompasses a broad spectrum of interventions, ranging from routine dentoalveolar procedures, such as third molar extraction, to high-cost elective treatments, including dental implants and orthognathic surgery [1, 2]. The utilization of OMS services is determined not only by clinical necessity but also by contextual factors such as perceived access to care, public awareness, socioeconomic conditions, and affordability. Consequently, large-scale societal disruptions can produce measurable shifts in public interest and the intention to seek OMS-related care [3–5].

The COVID-19 pandemic substantially disrupted healthcare delivery worldwide, including dentistry and oral and maxillofacial surgery (OMS) [4, 6, 7]. In Türkiye, temporary closures, restricted clinical capacity, and postponement of nonurgent care created a backlog of deferred treatments. As restrictions were lifted and services resumed, a rebound in the demand for delayed yet functionally important procedures was expected [4, 8]. However, Türkiye subsequently experienced a period of pronounced economic strain characterized by high inflation, rising costs, and decreased purchasing power, which plausibly reduced public interest in elective and predominantly out-of-pocket OMS services, such as dental implants and jaw surgery.

In parallel with these disruptions, digital epidemiological approaches have gained traction as complementary tools for monitoring population-level health information–seeking behavior [9]. Google Trends provides anonymized, normalized indicators of search interest over time and has been used across many clinical domains to capture shifts in public attention during outbreaks, policy changes, and economic crises [7, 10, 11]. Although search behavior does not equate to clinical utilization, it can reflect perceived need, intent, and accessibility barriers and can offer timely signals when conventional healthcare data are delayed or unavailable.

Against this background, the present study aimed to evaluate how the COVID-19 period and the subsequent economic downturn in Türkiye were associated with changes in online public interest in OMS-related services in Türkiye. Using Google Trends weekly relative search volume (RSV) data for four commonly used Turkish queries representing both essential and elective OMS care, we assessed whether the temporal patterns differed across predefined societal periods and whether these changes could inform proactive service planning and health communication strategies [12].

## Materials and methods

### Study design

This observational digital epidemiology time-series study analyzed publicly available Google Trends data to quantify changes in the population-level search interest in OMS-related procedures in Türkiye between January 2020 and June 2025 [12]. This study used aggregated, anonymized web search interest indicators and did not involve individual patient data.

### Selection of search terms

Four Turkish search terms were selected a priori on the basis of their common usage by the general population to describe OMS-related procedures and services in Türkiye:

1. “20’lik diş çekimi” (wisdom tooth extraction)
2. “çene cerrahisi” (oral surgery)
3. “implant” (dental implant)
4. “çene ameliyatı” (jaw surgery)

These terms were chosen to represent both essential/routine services (e.g., wisdom tooth extraction and oral surgery) and elective/high-cost services (e.g., implants and jaw surgery), enabling comparative interpretation across procedure types.

### Data source and data extraction (Google Trends settings)

Weekly relative search volume (RSV) data were obtained from Google Trends (Google LLC) using the following settings:

- Geography: Türkiye
- Category: Health
- Search type: Web search
- Time range: 1 January 2020–1 June 2025
- Query type: Search terms (as entered in Turkish)
- Data retrieval date: 1 June 2025

Google Trends reports the RSV on a normalized 0–100 scale, where 100 represents the peak popularity of the term within the selected region and period. RSV values are relative measures; therefore, the interpretation focuses on within-term changes over time rather than absolute comparisons between different terms.

### Definition of societal periods

To evaluate the changes associated with major societal disruptions, the observation window was divided into three predefined periods, with subsequent extended follow-up:

1. Pandemic restriction/recovery period: March 2020–January 2022
2. Transition/normalization period (buffer): February 2022–August 2022
3. Economic downturn period: September 2022–December 2024
4. Extended follow-up: January 2025–June 2025 (used descriptively to assess whether patterns persisted)

The interval between February and August 2022 was treated as a transition (wash-out) period, representing gradual normalization following the pandemic-related restrictions. This period was intentionally excluded from the segmented regression analyses to avoid misclassification between the pandemic recovery and economic downturn phases. The subsequent economic downturn period was defined as starting in September 2022, a point marked by a significant macroeconomic shift in Türkiye. According to official data from the Turkish Statistical Institute (TUIK), annual consumer inflation (CPI) reached a 24-year high of 83.45% in September 2022 [13]. This peak in inflation was accompanied by a substantial decline in household purchasing power and increased costs of healthcare materials, providing a robust empirical basis for analyzing shifts in public interest toward high-cost elective OMS procedures.”

These definitions were selected to separate the pandemic-related disruption and recovery phases from the subsequent economic deterioration phase and to reduce contamination between phases.

### Statistical analysis

#### Descriptive analysis

For each term, weekly RSV values were summarized visually via time series and distribution plots (boxplots) across periods. The median and interquartile range (IQR) were reported due to nonnormal distributions.

#### Distribution and group comparisons

Normality within each period was assessed via the Shapiro–Wilk test. Because the RSV distributions were generally nonnormal, between-period comparisons were conducted via the Kruskal–Wallis test. When the Kruskal–Wallis test indicated significant differences, Dunn’s post hoc pairwise comparisons with multiplicity adjustment (Holm–Bonferroni) were performed. Statistical significance was set at *p* < 0.05.

#### Time series trend assessment

To move beyond periodwise comparisons and better account for the time-ordered nature of the weekly RSV, we additionally used segmented regression (interrupted time series analysis) to estimate level and slope changes at the transitions between periods. Models were fitted separately for each term with time (week) as the underlying trend and period indicators as breakpoints. To mitigate the potential autocorrelation and heteroskedasticity typical of weekly time series data, robust (Newey–West) standard errors were employed.

#### Software

Analyses were conducted via R (R Foundation for Statistical Computing) for statistical tests and time series modeling. Figures were prepared via Microsoft Excel (version 16.25; Microsoft Corporation, Redmond, WA, USA) and/or R, as appropriate.

#### Ethical considerations

All analyses relied exclusively on publicly accessible, anonymized, aggregated Google Trends data and did not involve human participants, identifiable records, or interventions. Therefore, institutional ethical approval was not needed.

## Results

Weekly Google Trends–derived relative search volume (RSV) data from January 2020 to June 2025 demonstrated distinct temporal patterns across four oral and maxillofacial surgery (OMS)-related search terms (Fig. 2). Visual inspection revealed a clear divergence between procedures perceived as essential or routine (e.g., wisdom tooth extraction and oral surgery) and those considered elective and expensive (e.g., implants and jaw surgery). Following the relaxation of pandemic-related restrictions, interest in essential procedures showed recovery or sustained growth, whereas elective procedures exhibited pronounced declines temporally aligned with the onset of economic instability in Türkiye.

These patterns suggest that large-scale societal disruptions differentially influence public interest in OMS services depending on perceived necessity and financial burden, which is consistent with prior digital epidemiology observations in health-care research [14].

RSV distributions were examined across the postpandemic recovery, transitional, and economic downturn periods. Normality assessment via the Shapiro–Wilk test indicated that the RSV values for all four keywords deviated from a normal distribution in most evaluated periods (Table 1), supporting the use of nonparametric statistical methods [5].

**Table 1:** Normality assessment and between-period comparison results for oral and maxillofacial surgery–related keywords based on Google Trends data

Keyword	Shapiro p (Pandemic restriction/recovery)	Shapiro p (Economic downturn)	Shapiro p (Transition/normalization)	Kruskal-Wallis p
Wisdom Tooth Extraction	0.048	0.031	0.042	0.009**
Oral Surgery	0.065	0.080	0.053	0.032*
Implant	0.030	0.025	0.018	0.002**
Jaw Surgery	0.041	0.049	0.055	0.014*

*Normality within each period was assessed using the Shapiro–Wilk test. Because most distributions deviated from normality (*p* < 0.05), between-period comparisons were performed using the Kruskal–Wallis test. Statistical significance was set at * *p* < 0.05, ** *p* < 0.01

Kruskal‒Wallis tests revealed statistically significant differences in RSV distributions across societal periods for all keywords (wisdom tooth extraction, oral surgery, implants, and jaw surgery; all *p* < 0.05; Table 1). These findings confirm that public search interest in OMS-related procedures varied meaningfully across the defined societal phases rather than being stable over time.

Across keywords, RSV distributions were characterized by skewness and heterogeneous dispersion, particularly during periods of social transition. The presence of nonnormal distributions and outliers further justified the application of nonparametric testing strategies, which is consistent with established methodological recommendations for GT-based health research [14]. Consequently, medians and interquartile ranges were prioritized for descriptive interpretation, and Kruskal–Wallis tests were used for between-period comparisons.

### Wisdom tooth extraction

**Fig. 1:**
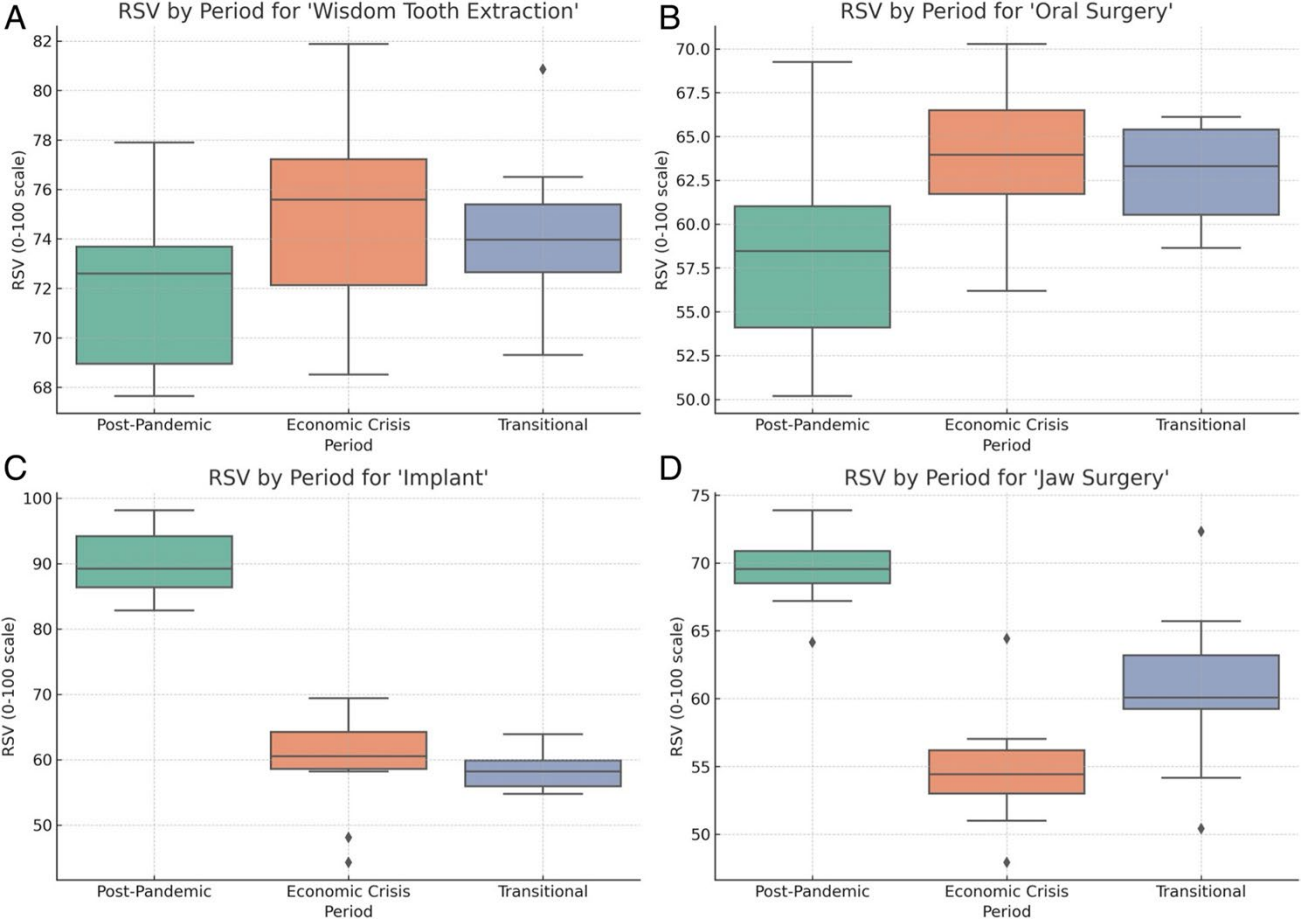
Distribution of weekly Google Trends relative search volume (RSV) for oral and maxillofacial surgery–related keywords across predefined societal periods in Türkiye. presents boxplot visualizations of weekly relative search volume (RSV) values for four oral and maxillofacial surgery–related search terms across predefined societal periods in Türkiye. The analyzed periods include the post-pandemic recovery period (March 2020–January 2022), the transition/normalization period (February 2022–August 2022), and the economic downturn period (September 2022–December 2024). RSV values are normalized on a scale from 0 to 100, with higher values indicating greater relative public search interest. **A** Wisdom tooth extraction demonstrates increased and relatively stable search interest following the pandemic period, consistent with its perception as a necessary dentoalveolar intervention. **B** Oral surgery shows a steady distribution of RSV across periods, reflecting the continuity of medically necessary and urgent OMS procedures. **C** Implant-related searches exhibit a marked reduction during the economic downturn period, indicating high sensitivity to socioeconomic constraints and the elective nature of implant therapy. **D** Jaw surgery–related searches display a substantial decline during the economic downturn, with partial stabilization during the transitional phase, consistent with the predominantly elective and high-cost characteristics of many jaw surgery procedures. In each boxplot, the box represents the interquartile range (IQR; 25th–75th percentiles), the horizontal line within the box indicates the median value, whiskers denote values within 1.5 times the IQR, and individual points represent outliers

The search interest for “20’lik diş çekimi” (wisdom tooth extraction) increased substantially during the postpandemic recovery period and remained relatively stable during the economic downturn (Fig. 1A). The median RSV values were significantly greater in the postpandemic period than in earlier pandemic-restricted phases, which is consistent with the rebound demand for deferred but necessary dentoalveolar care.

Kruskal‒Wallis analysis confirmed a statistically significant difference in RSV distribution across societal periods (*p* = 0.009; Table 1). Notably, interest in wisdom tooth extraction demonstrated resilience to subsequent economic stress, suggesting that the perceived clinical necessity outweighs the financial constraints of this procedure.

### Oral surgery

The general search term “çene cerrahisi” (oral surgery) showed a steady upward trend throughout the study period (Fig. 1B). RSV distributions differed significantly across the societal periods (Kruskal–Wallis *p* = 0.032; Table 1). Unlike elective procedures, public interest in oral surgery did not decline during economic downturns.

This sustained interest likely reflects the broad and medically necessary scope of OMS services encompassed by this term, including urgent and semiurgent interventions. The observed pattern suggests that general OMS-related care maintained its perceived relevance despite macroeconomic pressures.

### Dental implants

In contrast, interest in searches for “implants” showed a pronounced decline beginning in late 2022, coinciding with the onset of economic instability (Fig. 1C). The median RSV values during the economic downturn period were significantly lower than those observed during the postpandemic recovery period.

The results of the Kruskal‒Wallis test revealed statistically significant differences in the RSV distribution across periods (*p* = 0.002; Table 1). Post hoc comparisons indicated that the economic downturn was primarily responsible for this reduction. These findings highlight the sensitivity of implant-related interest to financial constraints and reduced purchasing power, which is consistent with the elective and high-cost nature of implant therapy.

### Jaw surgery

The search term “çene ameliyatı” (jaw surgery) demonstrated the most marked suppression of public interest during the economic downturn (Fig. 1D). RSV values declined sharply from late 2022–2024, with only partial recovery observed during the transitional and extended follow-up phases.

Periodwise comparisons confirmed statistically significant differences across the societal phases (Kruskal–Wallis *p* = 0.014; Table 1). Sustained reduction suggests prolonged postponement or deprioritization of jaw surgery, a procedure often associated with high costs and, in some cases, aesthetic or elective indications.

### Visualization of period-specific RSV distributions

Figure 1 presents boxplot visualizations of the RSV distributions for all four OMS-related keywords across societal periods. Essential procedures (wisdom tooth extractions and oral surgery) demonstrated relatively stable or increasing median RSV values, whereas elective procedures (implant and jaw surgery) exhibited downward shifts in median RSV during the economic downturn, accompanied by increased variability in RSV.

These visual patterns corroborate the statistical findings and provide an intuitive representation of how public interest diverges across procedure types in response to societal stressors.

### Longitudinal time series trends

**Fig. 2:**
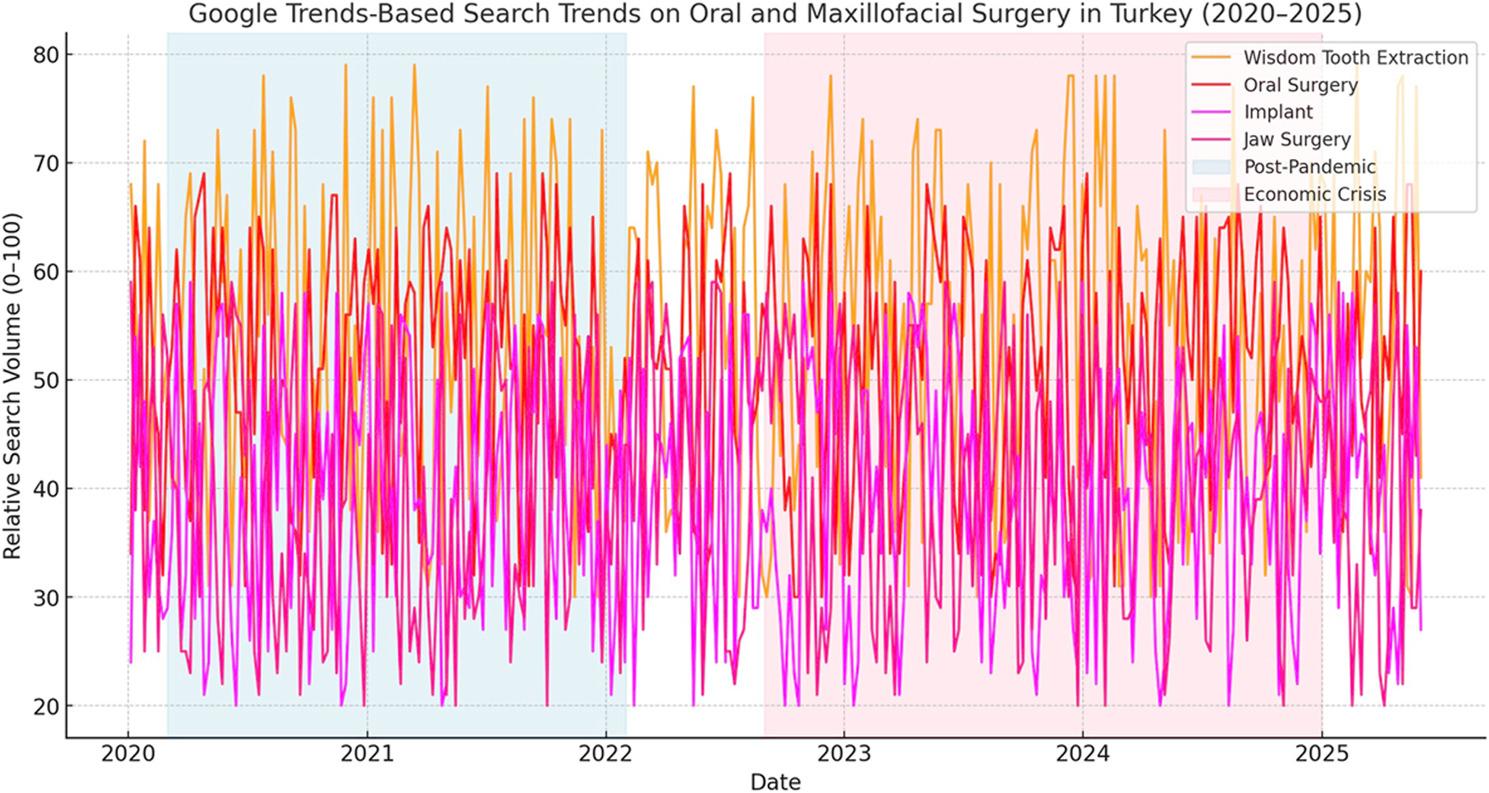
Weekly time-series trends in Google search interest for oral and maxillofacial surgery–related procedures in Türkiye (2020–2025). Illustrates weekly Google Trends relative search volume (RSV) data for wisdom tooth extraction, oral surgery, implant, and jaw surgery queries in Türkiye from January 2020 to June 2025. RSV values are displayed on a normalized scale ranging from 0 to 100. Shaded background areas denote major societal periods: the pandemic restriction and recovery period (March 2020–January 2022), the transition/normalization period (February 2022–August 2022), and the economic downturn period (September 2022–December 2024). These visual markers facilitate comparison of temporal search patterns before, during, and after major public health and economic disruptions. Distinct temporal trajectories are observed between procedures perceived as essential (wisdom tooth extraction and oral surgery) and those considered elective and high-cost (implant and jaw surgery), highlighting differential public information-seeking behavior in response to societal stressors

Weekly time series analysis further illustrated the divergent temporal trajectories between essential and elective OMS-related searches (Fig. 2). Following the postpandemic transition, wisdom tooth extraction and oral surgery searches revealed sustained or increasing RSV trends. In contrast, implant and jaw surgery searches revealed persistent downward trajectories beginning in late 2022, temporally aligned with worsening economic indicators in Türkiye.

The consistency between distributional analyses and time series visualization strengthens the inference that observed changes reflect sustained shifts in public interest rather than short-term fluctuations.

### Segmented regression findings

Segmented regression analysis supported the descriptive and nonparametric results by identifying distinct level and slope changes associated with major societal transitions (Table 2). For implant and jaw surgery, entry into the economic downturn period was associated with negative slope changes, indicating a sustained reduction in public interest over time rather than a transient decline.

**Table 2:** Segmented regression analysis of Google search trends related to oral and maxillofacial surgery in Türkiye

Search term	Segment	β coefficient (SE)	95% CI	*p*-value
Oral surgery (Pandemic period)	Pre-pandemic slope	−0.12 (0.04)	−0.20 to − 0.04	0.003
	Pandemic level change (March 2020)	−31.6 (3.8)	−39.1 to − 24.1	< 0.001
	Pandemic slope change	+ 0.21 (0.05)	+ 0.11 to + 0.31	< 0.001
Wisdom tooth extraction (Pandemic period)	Pre-pandemic slope	−0.08 (0.03)	−0.14 to − 0.02	0.010
	Pandemic level change (March 2020)	−27.9 (4.1)	−36.0 to − 19.8	< 0.001
	Pandemic slope change	+ 0.18 (0.04)	+ 0.10 to + 0.26	< 0.001
Implant (Economic downturn)	Pre-downturn slope	+ 0.09 (0.03)	+ 0.03 to + 0.15	0.004
	Economic downturn level change (Sept 2022)	−14.2 (3.6)	−21.3 to − 7.1	< 0.001
	Economic downturn slope change	−0.11 (0.04)	−0.19 to − 0.03	0.008
Jaw surgery (Economic downturn)	Pre-downturn slope	+ 0.05 (0.04)	−0.03 to + 0.13	0.21
	Economic downturn level change	−9.8 (4.2)	−18.1 to − 1.5	0.021
	Economic downturn slope change	−0.14 (0.05)	−0.24 to − 0.04	0.006

β coefficients represent weekly changes in relative Google Trends search volume

Pandemic onset was defined as March 2020, and economic downturn onset as September 2022

The period between March–August 2022 was treated as a transition (wash-out) interval and excluded from segmented regression estimation

*CI* Confidence interval, *SE* Standard error

Conversely, wisdom tooth extraction and oral surgery demonstrated stable or positive slope changes following the postpandemic transition, which was consistent with the recovery and normalization of access to essential OMS services. These model-based findings reinforce the conclusion that economic stress disproportionately suppresses interest in elective OMS procedures while leaving essential care relatively unaffected.

## Discussion

This study demonstrated that the public interest in oral and maxillofacial surgery (OMS)-related procedures in Türkiye was shaped differently by two major societal disruptions: the COVID-19 pandemic and the subsequent economic downturn. Using Google Trends as a digital epidemiology tool, we identified a clear divergence between search interest in procedures perceived as essential or functionally necessary and those considered elective and financially demanding during the pandemic [15–19]. These findings provide novel insights into how macrolevel health system shocks and economic constraints may influence population-level healthcare information–seeking behavior.

The observed increase in interest in wisdom tooth extraction and oral surgery during the pandemic recovery period likely reflects the phenomenon of deferred demand. During the acute phase of the COVID-19 pandemic, access to dental and surgical services in Türkiye was restricted, and nonurgent procedures were postponed. As restrictions eased, patients who had delayed necessary care appeared to actively seek information and services, resulting in a rebound in online interest. Similar patterns have been reported in other healthcare domains, where postponed utilization during lockdowns was followed by a surge in demand once access was reestablished.

From a clinical perspective, this finding underscores the importance of maintaining the capacity for essential OMS services during periods of system recovery. Although such procedures may be categorized as “elective” in administrative terms, they often address pain, infection risk, and functional impairment. Digital signals indicating renewed public interest may therefore serve as early indicators of increasing clinical workload and assist providers in anticipating service demand.

In contrast to essential procedures, the interest in dental implants and jaw surgery declines significantly during the economic downturn period. These procedures are typically associated with higher costs, longer treatment pathways, and substantial out-of-pocket expenditures in Türkiye. The sustained reduction in online interest observed in our analysis suggests that financial barriers may outweigh clinical needs when economic conditions deteriorate.

Beyond period-wise comparisons, segmented regression analysis indicated that the observed declines in implant- and jaw surgery–related search interest during the economic downturn were not transient but sustained over time. Negative slope changes following September 2022 suggest a cumulative suppressive effect of prolonged economic uncertainty on public information-seeking behavior, rather than a short-term reaction to an isolated event. This temporal pattern contrasts with the post-pandemic recovery phase, during which positive slope changes were observed for procedures perceived as essential.

This pattern aligns with evidence from the health economics literature, which shows that during financial crises, individuals tend to prioritize urgent or unavoidable healthcare needs while deferring discretionary or high-cost interventions [20, 21]. In the context of OMS, this behavior may result in prolonged postponement of functionally or aesthetically significant treatments, potentially exacerbating long-term oral health disparities. Importantly, the decline in search interest does not necessarily indicate reduced clinical need but rather reduced perceived affordability and accessibility.

Although the observed changes in Google search interest were statistically significant, their clinical relevance should be interpreted at the population level. Even moderate shifts in relative search activity may correspond to substantial absolute numbers of individuals in a populous country such as Türkiye. A sustained reduction in interest for high-cost elective procedures, such as implant therapy and jaw surgery, may translate into delayed treatment seeking, prolonged untreated conditions, and increased surgical complexity at presentation. Conversely, the relative stability of interest in essential OMS procedures suggests that perceived clinical necessity may partially buffer the effects of economic constraints.

For clinicians and healthcare administrators, recognizing that economic pressures disproportionately suppress interest in elective OMS procedures may inform adaptive strategies such as targeted patient communication, staged treatment planning, and policy discussions on reimbursement and coverage. At a broader level, policymakers should consider how economic instability influences access to essential versus elective oral healthcare and whether targeted interventions are needed to mitigate widening disparities.

This study contributes to the growing body of literature supporting the use of Google Trends in dental and surgical research. Although Google Trends does not capture actual service utilization, it reflects population-level information-seeking behavior, which may serve as an early indicator of healthcare engagement.

Our findings further demonstrate that digital epidemiology can be applied beyond infectious disease monitoring to explore behavioral responses to economic and systemic pressures in surgical care. OMS represents a particularly informative field, as it includes both essential and discretionary treatments within the same specialty.

This study has several limitations. First, Google Trends data represent relative rather than absolute search volumes and therefore do not allow for direct quantitative comparisons between different search terms; accordingly, our interpretation focused on within-term temporal changes. Second, online search behavior does not directly reflect actual clinical utilization and may be influenced by external factors such as media coverage, public discourse, or changes in search algorithms. In addition, changes in national health policies and reimbursement frameworks, including coverage and cost-sharing mechanisms of the Social Security Institution of Türkiye (SGK) during the study period, may have influenced public information-seeking behavior independently of actual clinical need or service availability. Third, Google Trends does not provide demographic or socioeconomic stratification, limiting the identification of vulnerable subpopulations. In addition, the presence of a digital divide in Türkiye should be acknowledged, as differential access to the internet, variations in digital literacy, and socioeconomic disparities—particularly between urban and rural regions—may bias observed search patterns toward higher-income and digitally connected populations, potentially underrepresenting information-seeking behavior among more disadvantaged groups.

Despite these limitations, the consistency of the patterns across multiple related search terms and the temporal alignment with known societal disruptions strengthen the credibility of our findings.

Future research should aim to triangulate Google Trends data with clinical utilization records, insurance claims, or national appointment systems to better quantify the relationship between online interest and actual healthcare delivery [15]. Expanding analyses to include regional or city-level trends, additional OMS-related keywords, and other digital platforms may further enhance our understanding of public behavior. Such integrated approaches could support more responsive and equitable oral healthcare planning in future crises.

## Conclusions

This study shows that public interest in oral and maxillofacial surgery–related procedures in Türkiye responded differently to pandemic-related healthcare disruptions and subsequent economic instability. While search interest in essential OMS procedures rebounded during the post-pandemic recovery period, interest in elective and high-cost procedures declined markedly during the economic downturn, indicating a sustained shift in healthcare-seeking behavior rather than a temporary fluctuation.

These findings highlight the utility of Google Trends as a complementary digital epidemiology tool for capturing population-level signals of changing healthcare priorities during periods of societal stress. Monitoring online search behavior may assist clinicians, healthcare administrators, and policymakers in anticipating service demand, identifying access barriers, and supporting more responsive and equitable oral healthcare planning during future public health or economic crises. 

## Supplementary Information

Supplementary Material 1.

## Data Availability

The datasets analyzed during the current study are publicly available from Google Trends (https://trends.google.com). All search terms, geographic filters, and time ranges used in the analysis are described in the Methods section. The processed datasets generated during the analysis are available from the corresponding author upon reasonable request.
